# Effects of Pandemics on Corporate Innovation: Evidence From the Chinese Firms

**DOI:** 10.3389/fpubh.2021.780348

**Published:** 2021-11-04

**Authors:** Ci Zhang, Yilin Hu, Leping Huang, Yajie Huang

**Affiliations:** ^1^School of Business, Macau University of Science and Technology, Taipa, Macao SAR, China; ^2^Johnson & Johnson Medical (Shanghai) Ltd., Shanghai, China; ^3^School of Foreign Languages, Tianjin University of Commerce, Tianjin, China; ^4^School of Economics, Tianjin University of Commerce, Tianjin, China

**Keywords:** COVID-19 pandemic, post-COVID-19 era, pandemics discussion index, corporate innovation, Chinese firms, Blundell-Bond estimations

## Abstract

This paper examines the effects of the pandemics-related uncertainty on corporate innovation in Chinese firms. For this purpose, the recent uncertainty measure of pandemics, the Pandemics Discussion Index (PDI), is used. The findings from the fixed-effects estimations show the negative impact of the PDI on corporate innovation. Government subsidies, operation profits, and total exports also positively affect corporate innovation. In addition, firms' management efficiency promotes corporate innovation. These results hold when the Blundell-Bond estimations are utilized to address potential endogeneity. Various robustness analyses, such as considering the lagged PDI and the lagged controls, are also conducted. Consequently, the main results remain robust. Thus, this paper provides novel and robust evidence on the negative impact of pandemics on Chinese firms' corporate innovation behavior.

## Introduction

Corporate innovation is one of the main sources of economic growth (1, 2). It also provides the efficient reallocation of sources, and thus, it can promote economic performance (3, 4). Corporate innovation promotes productivity and productivity gains spillovers to the whole economy in general (5, 6). Corporate innovation is also one of the determinants of upgrading export quality, especially in developing economies like China (7). Following the reform in 1978, the Chinese economy has rapidly grown with an average growth rate of around 10% over four decades (8). This great growth performance has taken attention from academia and policymakers (9). Several papers observe that innovation is the key aspect of this solid economic performance in China (10, 11). Corporate innovation also leads to a successful transition from fossil fuels to clean technology (12). Therefore, the sustainability of the corporate innovation process is vital for economic growth sustainability in China.

There are various determinants of corporate innovation, such as domestic credits (13, 14), financial development (15), institutional quality (16, 17), stock market development (18), and trust (19). Recently, several papers show that uncertainty shocks significantly affect corporate innovation. We expect that uncertainty shocks negatively affect the level of investments since they increase financing costs of innovation. At this juncture, policy uncertainty decreases the equity risk premium since policy uncertainty creates a political risk (20–22). Uncertainty may also affect capital expenditures are due to the fluctuations in bond prices, which are a significant determinant of financing costs of innovation (23). Various papers show that uncertainty shocks negatively affect corporate investments in general, including new investments and innovation expenditures in particular (24–35). Refer also to Bernanke (36), Caballero (37), Carruth et al. (38), and Rodrik (39) for the early empirical and theoretical papers in the literature.

This paper aims to examine the effects of the pandemics-related uncertainty on corporate innovation in Chinese firms. For this purpose, we use the recent uncertainty measure of pandemics: The Pandemics Discussion Index (PDI). It is important to note that previous papers on the effects of uncertainty shock on corporate innovation mostly focus on the regional or the national-level data. Therefore, there is a gap in the empirical literature for analyzing the effects of uncertainty shocks across corporate innovation by the firm-level data. Indeed, uncertainties can significantly affect firms' new investment decisions, increasing lending costs. This research is the first paper to use the PDI as the determinant of corporate innovation in China to the best of our knowledge. In this paper, we find a negative impact of the PDI on corporate innovation. In addition, government subsidies, operation profits, management efficiency, and total exports positively affect corporate innovation. These results remain valid when we utilize the Blundell-Bond estimations; thus, we address potential endogeneity. We also conduct various robustness analyses, such as considering the lagged PDI and the lagged controls. Therefore, we provide novel and robust evidence on the negative impact of pandemics on Chinese firms' corporate innovation behavior.

The remaining parts of the study are structured as follows. Section Literature Review reviews the previous papers on literature examining the determinants of corporate innovation in developing and developed economies, including China. Section Dataset and the Estimated Models explains the dataset of the Chinese firms, estimated empirical models, and method of the pandemics discussion index in China. Section Empirical Findings discusses the findings of the fixed-effects and the Blundell–Bond estimations for the current and the lagged models. Section Concluding Remarks provides the concluding remarks.

## Literature Review

Various papers are examining the determinants of corporate innovation both in developing and developed economies, including China. Uncertainty measures are also included as the potential driver of corporate innovation. For instance, Wei et al. (11) indicate that the sustainable growth performance of the Chinese economy depends on productivity gains and domestic innovations. The authors use the expenditures on research and development and patent applications and observe that the innovation level in China has increased during the last decades. The authors find that the higher real wages and the growing domestic markets are the leading determinants of the growth of corporate innovation in the Chinese economy. More interestingly, the authors obtain the evidence for misallocation of resources during the innovation decisions; that is, the state-owned corporations are taking higher subsidies from the government; however, the innovation growth rate of the private firms are higher than the state-owned corporations. Following these results, the authors conclude that the sustainability of corporate innovation in the Chinese economy depends on mitigating the level of resource misallocation in the economy. Rong et al. (40) examine the Chinese firms' determinants of corporate innovation (measured by patent applications) from 2002 to 2011. The authors observe that institutional investors are positively related to corporate innovation. More specifically, the positive impact of institutional investors on patent applications comes from mutual funds. In addition, market competition increases corporate innovation. Furthermore, private-owned firms have a higher number of patent applications than state-owned firms. These results are also robust to consider different measures of corporate innovation.

Similarly, Kroll and Kou (41) find that there are negative effects of the state-owned firms (both central and local governments) on the number of firms' patent applications. Meng et al. (19) investigate the effects of trust on corporate innovation at the firm-level data in 72 countries. The authors find that a higher level of trust and intellectual property rights causes higher R&D expenditures (investments) from 1992 to 2016. The authors discuss that greater trust and intellectual property rights in a country can suppress information asymmetries, and this issue decreases transaction costs. Thus, there will be fewer financial constraints for innovation. Song et al. (42) indicate that innovation in the Chinese manufacturing sector relates to the global value chain participation and labor division. Theoretically speaking, the role of the COVID-19 on innovation can be negative or positive. However, the COVID-19 pandemic hurts the global value chain participation with the trading partners, and therefore the authors conclude that the COVID-19 pandemic negatively affects corporate innovation in the Chinese manufacturing sector. Given this backdrop, we suggest that the pandemics are negatively related to innovation.

There are also previous papers to examine the effects of uncertainty shocks on corporate innovation. These papers mostly use the index of economic uncertainty index to capture the effects of uncertainty shocks. For example, Wang et al. (43) use the data of the Chinese firms to study the effects of economic policy uncertainty on R&D investments. The authors show that economic policy uncertainty decreases the R&D investments in politically connected firms, and the significant impact comes from the government subsidies. Contrarily, He et al. (44) indicate that the economic policy uncertainty positively impacts corporate innovation in the Chinese economy from 2000 to 2017. Economic policy uncertainty has a greater positive impact on state-owned firms. In a similar vein, Guan et al. (45) investigate the effects of the Chinese economic policy uncertainty (CEPU) on corporate innovation in Chinese firms and find that the CEPU positively affects corporate innovation. Market competition is a vital mechanism for enhancing corporate innovation during times of higher CEPU.

On the other hand, Xu (46) investigates the effects of economic policy uncertainty shocks on the United States firms' innovation activities with the cost of capital. The author observes that economic policy uncertainty increases firms' cost of capital, and this issue leads to a lower level of innovation expenditures. Cui et al. (47) find that economic policy uncertainty decreases corporate investments in Chinese firms from 2007 to 2017. Finally, Lou et al. (48) examine the effects of economic policy uncertainty on firm innovation in China's A-share listed firms from 2001 to 2017. The authors observe that economic policy uncertainty decreases firms' innovation investments.

Overall, according to the literature review, we observe that there are various papers in the empirical literature to investigate the effects of uncertainty shocks on corporate innovation in China. Most of these studies use the indices of economic policy uncertainty. To the best of our knowledge, there is no paper in the empirical literature to examine the pandemics-related uncertainty measures on corporate innovation in Chinese firms.

## Dataset and the Estimated Models

### Dataset of the Chinese Firms

There are several issues regarding modeling the determinants of corporate innovation at the firm-level data. The first issue is that the ordinary least squares (OLS) estimations have the potential problem of simultaneity bias (49). Therefore, one should include the fixed effects of the firms' industry (sector) and regions (province). The second issue is that the level of corporate innovation can be related to the level of corporate innovation in the previous years. In other words, the lagged corporate innovation can determine its current level or the level in the next period. The third issue is that there can be a reverse causality issue, given that there will be various determinants of corporate innovation. Therefore, it is better to use both the current and lagged models when the determinants of corporate innovation will be examined. Our empirical models and estimation procedures consider these three potential issues in the empirical examination.

We focus on the firm-level data from 2000 to 2013, which considers the Chinese Industrial Enterprise Database with the MOC List of Chinese firms. We combine the dataset from 2000 to 2007 and the newer dataset over the period 2008–2013. We merged two datasets and filtered missing indicators following the methodology of Brandt et al. (50). We delete the samples with zero and negative values of the control variables in the estimations. If there is a missing value in one indicator, we also skip these firms or years. In addition, outlier observations, such as total assets < fixed assets or liquid assets, are cleaned from the dataset. There is also some misinformation in the dataset, such as age <0, and we remove these observations. We also focus on the firms, which have an operational revenue higher than 1,000 RMB. Note that we focus on the real RMB prices, and the realization of the prices are based on the producer price index deflator of the gross domestic product. The baseline year is 2000 in calculating the real RMB prices. At this stage, we use 282,556 observations from the 44,337 firms for the empirical analyses.

### Estimated Empirical Models

According to the theories and previous empirical papers [e.g., (34, 51–54)], which are previously discussed, indicate that the effects of uncertainty shocks on corporate innovation should be negative. Following this theoretical background, we indicate that pandemics-related uncertainty should negatively affect corporate innovation. Therefore, we can write down the following empirical models:


(1) $$\begin{array}{l} innov_{i,t}=\beta_{0}+\beta_{1}china\text{\_}pdi_{i,t}+\beta_{2}X_{i,t}+\upsilon_{i}+\mu_{i}+\varepsilon_{i,t} \end{array}$$

(2) $$\begin{array}{l} innov_{i,t}=\beta_{3}+\beta_{4}china\text{\_}pdi_{i,t-1}+\beta_{5}X_{i,t}+\upsilon_{i}+\mu_{i}+\varepsilon_{i,t} \end{array}$$

(3) $$\begin{array}{l} innov_{i,t}=\beta_{6}+\beta_{7}china\text{\_}pdi_{i,t}+\beta_{8}X_{i,t-1}+\upsilon_{i}+\mu_{i}+\varepsilon_{i,t} \end{array}$$

(4) $$\begin{array}{l} innov_{i,t}=\beta_{9}+\beta_{10}china\_pdi_{i,t-1}+\beta_{11}X_{i,t-1}+\upsilon_{i}+\mu_{i}\\ \;+\;\varepsilon_{i,t} \end{array}$$

(5) $$\begin{array}{l} innov_{i,t}=\alpha_{0}+\alpha_{1}innov_{i,t-1}+\alpha_{2}china\_pdi_{i,t}+\alpha_{3}X_{i,t}+\upsilon_{i}\\ \;+\;\mu_{i}+\varepsilon_{i,t} \end{array}$$

(6) $$\begin{array}{l} innov_{i,t}=\alpha_{4}+\alpha_{5}innov_{i,t-1}+\alpha_{6}china\_pdi_{i,t-1}+\alpha_{7}X_{i,t}\\ \;+\upsilon_{i}+\mu_{i}+\varepsilon_{i,t} \end{array}$$

(7) $$\begin{array}{l} innov_{i,t}=\alpha_{8}+\alpha_{9}innov_{i,t-1}+\alpha_{10}china\_pdi_{i,t}+\alpha_{11}X_{i,t-1}\\ \;+\;\upsilon_{i}+\mu_{i}+\varepsilon_{i,t} \end{array}$$

(8) $$\begin{array}{l} innov_{i,t}=\alpha_{12}+\alpha_{13}innov_{i,t-1}+\alpha_{14}china\_pdi_{i,t-1}+\alpha_{15}X_{i,t-1}\\ \;+\;\upsilon_{i}+\mu_{i}+\varepsilon_{i,t} \end{array}$$

As discussed before, there can be a reverse causality issue between the explanatory variables and the dependent variable; therefore, we use the lagged variables.

Where *innov*_*i,t*_ and *innov*_*i,t*−1_ are the current and the lagged corporate innovation, where *i* denotes the firm, and *t* indicates the year. *china*_*pdi*_*i,t*_ and *china*_*pdi*_*i,t*−1_ are the current and the lagged pandemics-related uncertainty index (pandemic discussion index) in China, *X*_*i,t*_ and *X*_*i,t*−1_ are the following control variables in the current and the lagged forms: *profit* is the firm's profit, *expo_int* is the firm's exports intensity, *fin_cons* is the firm's financial constraints, *state_owner* is the firm's ownership, *mana_eff* is the firm's management efficiency, *gov_subs*, is the government subsidies to firm, *valuad_pro* is the value-added productivity per employee, and *firm_age* is the firm's age. Finally, υ_*i*_ is the industry (sector) fixed-effects, μ_*i*_ is the province (region) fixed-effects, and ε_*i,t*_ represents the stochastic disturbance error term.

We estimate the models in Equations (1)–(8) using the fixed-effects and the system GMM estimations of Blundell and Bond (55). Similar to the differenced GMM estimations of Arellano and Bond (56), the system GMM estimation technique uses the lagged dependent variable. Therefore, the stochastic disturbance error term is subjected to autocorrelation. At this stage, we use the robust standard errors provided by Windmeijer (57). In the system GMM estimations, we need to find the first-order autocorrelation following the AR(1) test results. However, we need to obtain no significant second-order autocorrelation following the AR(2) test results. We also need to utilize the Sargan test for checking over-identifying restrictions. We need to reject the null hypothesis that all instrumental variables are not correlated to the stochastic disturbance error term. Thus, we can conclude that there is no over-identifying restriction problem in the system GMM estimations. We run the xtabond2 estimation procedure in Stata following Roodman's (58) suggestions under this backdrop.

### Calculating the Pandemics Discussion Index in China (China_ PDI)

This measure is the aggregate index of discussion about pandemics in a given country, China in our case. The PDI is calculated by Ahir et al. (59), counting the number of times a word related to pandemics is written in the Economist Intelligence Unit country reports. A higher value indicates higher discussion about pandemics and vice versa, and the index captures the pandemics-related uncertainty. The PDI index extends the seminal measure of economic policy uncertainty (EPU) introduced by Baker et al. (25). Note that the PDI measure is provided by quarterly data. Given that our firm-level data have annual frequency data, we take a simple average to convert the quarterly PDI data into the annual data frequency as follows:


(9) $$\begin{array}{l} CHINA\text{\_}PDI_{t}=\frac{\overset{4}{\underset{m=1}{\sum }}CHINA\text{\_}PDI_{q}}{4} \end{array}$$

**Figure 1:**
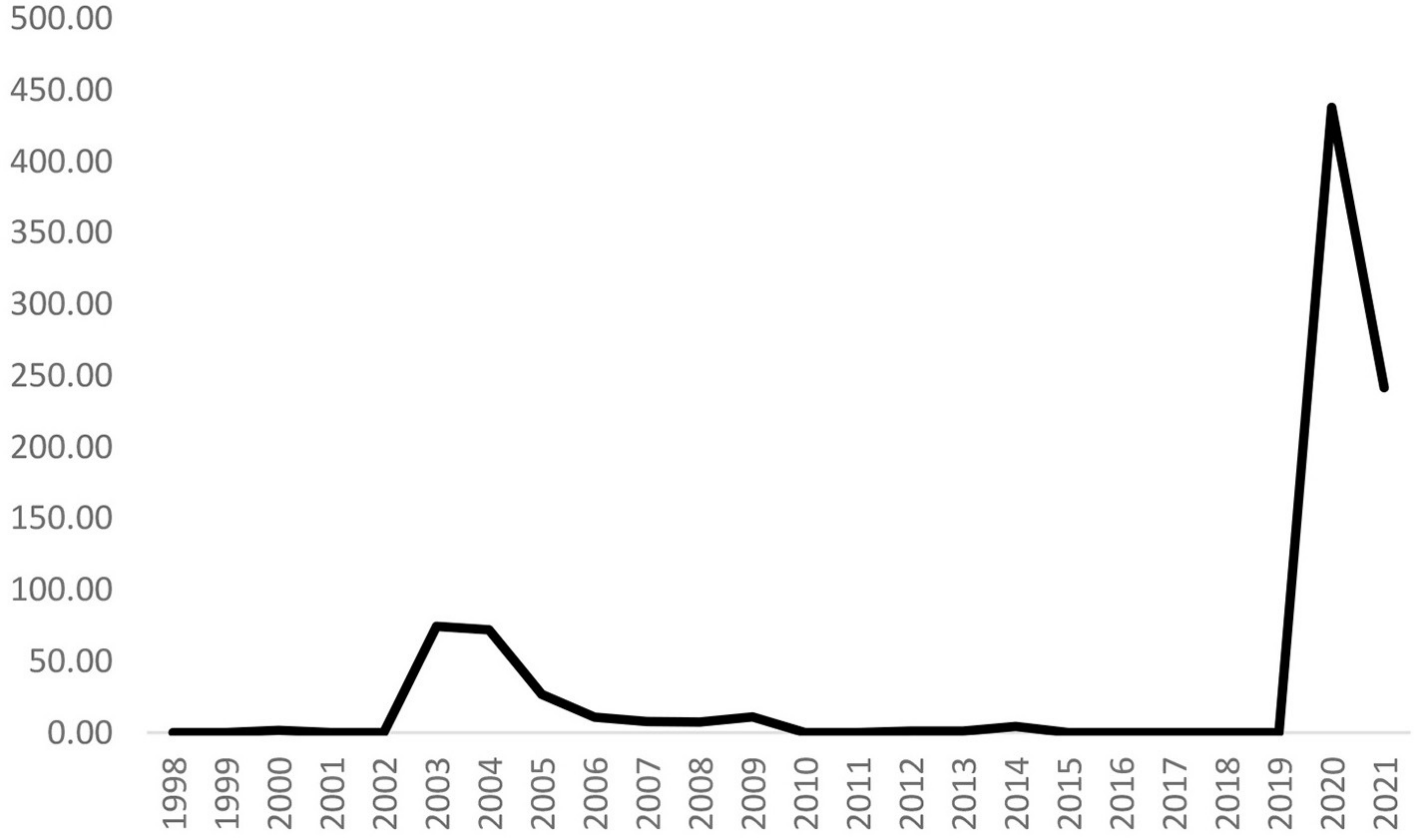
Pandemics discussion index in China (Annual, 1998–2021Q2). Data source: https://worlduncertaintyindex.com/data/, based on Ahir et al. (59).

In Equation (9), *CHINA*_*PDI*_*q*_ is the index of pandemics discussion index in China in four quarters given a year, and *CHINA*_*PDI*_*t*_ is the annual value of the index when we take the average value of pandemics discussion index in China. Figure 1 depicts the annual frequency pandemics discussion index in China from 1998 to 2021Q2.

Furthermore, Table 1 represents the indicators used in the empirical analyses and their definitions. Note that indicators are calculated with the current (nominal) prices in the Chinese RMB.

**Table 1:** Labels and definitions of indicators in the estimations.

**Label**	**Indicator**	**Definition**
innov	Firm's innovation level	The ratio of sales revenue of newly introduced products in total sales revenue
china_pdi	Pandemics discussion index in China	Annual values based on averages of quarterly values
profit	Firm's profit	Operation profits relative to total sales revenue
expo_int	Firm's exports intensity	Total exports relative to total sales revenue
fin_cons	Firm's financial constraints	Expenses on interest relative to the value of fixed assets
state_owner	Firm's ownership	Share of state ownership in total paid-in capital
mana_eff	Firm's management efficiency	Primary operation revenues relative to the value of total assets
gov_subs	Government subsidies to firm	Government subsidies relative to total sales revenue
valuad_pro	Value-added productivity per employee	Log (operating profit added to salaries and payroll expense per employee)
firm_age	Firm's age	Log (based year—year of establishment of the firm +1)

*Indicators are calculated with the current (nominal) prices in the RMB*.

Table 2 summarizes descriptive statistics indicators used in the empirical analyses, including the mean, the standard deviations, the minimum values, the maximum values, and observations.

**Table 2:** Summary of descriptive statistics.

**Indicator**	**Mean**	**Std. Dev**.	**Min**.	**Max**.	**Obs**.
innov	0.010	0.076	0.000	0.959	282,556
china_pdi	37.44	99.68	0.000	437.9	282,556
profitr	0.064	0.161	−82.41	120.4	282,556
expo_int	0.159	0.396	0.000	42.31	282,556
fin_cons	0.174	0.394	0.000	13.19	282,556
state_owner	0.451	0.164	0.000	1.000	239,542
mana_eff	0.704	0.826	0.000	3.871	282,556
gov_subs	0.050	0.171	0.000	1.448	282,556
valuad_pro	5.546	1.133	0.000	16.26	282,556
firm_age	11.14	10.021	0.000	414.0	282,556

## Empirical Findings

### Findings of the Baseline Fixed-Effects Estimations

Table 3 provides the findings of the fixed-effects estimations with the current controls, where the dependent variable is the corporate innovation (*innov*_*t*_). The industry fixed-effects and the province fixed-effects are included in the fixed-effects estimations.

**Table 3:** Results of the Benchmark fixed-effects estimations (current controls).

**Dependent variable: innov_**t**_**	**Full-sample**	
china_pdi _t_	−0.690^***^ [−2.72]	–
china_pdi _t−1_	–	−0.573^**^ [−2.51]
profit _t_	0.818^***^ [3.84]	0.843^***^ [3.76]
expo_int _t_	0.006^***^ [2.58]	0.005^***^ [2.64]
fin_cons _t_	−0.037 [−1.35]	−0.041 [−1.41]
state_owner _t_	0.367 [1.09]	0.356 [1.02]
mana_eff _t_	0.755^***^ [6.26]	0.783^***^ [6.52]
gov_subs _t_	0.053^***^ [5.01]	0.058^***^ [5.22]
valuad_pro _t_	0.002 [1.39]	0.002 [1.30]
firm_age _t_	0.011 [1.13]	0.012 [1.25]
Constant term	Yes	Yes
Industry fixed-effects	Yes	Yes
Province fixed-effects	Yes	Yes
Observation	239,542	205,196
R-squared	0.192	0.195
F-statistics	634.2^***^	651.2^***^

*The t-statistics are in brackets*.

*** *p < 0.01 and*

** *p < 0.05*.

The left column uses the current pandemic discussion index in China (*china_pdi*_*t*_), and the right column considers the lagged pandemic discussion index in China (*china_pdi*_*t*−1_). Both measures of the pandemics-related uncertainty are negatively related to corporate innovation. Note that China's current pandemic discussion index is statistically significant at the 1% level, and the lagged pandemic discussion index in China is statistically significant at the 5% level.

When we look at the control variables, the operation profits (*profit*_*t*_), the total exports (*expo_int*_*t*_), the management efficiency (*mana_eff*_*t*_), and the government subsidies (*gov_subs*_*t*_) increase the level of corporate innovation. All of these variables are statistically significant at the 1% level. The state ownership (*state_owner*_*t*_), the value-added productivity (*valuad_pro*_*t*_), and the firm age (*firm_age*_*t*_) are also associated with the corporate innovation; however, their coefficients are statistically insignificant. On the other hand, financial constraints (*fin_cons*_*t*_) are negatively related to the level of corporate innovation. However, the related coefficients are also statistically insignificant.

### Findings of the Further Fixed-Effects Estimations

Table 4 reports the results of the fixed-effects estimations with the lagged controls, and the dependent variable is the corporate innovation (*innov*_*t*_). Again, the industry fixed-effects and the province fixed-effects are included in the fixed-effects estimations.

**Table 4:** Results of the Benchmark fixed-effects estimations (lagged controls).

**Dependent variable: innov_**t**_**	**Full-sample**	
china_pdi _t_	−0.715^***^ [−2.78]	–
china_pdi _t−1_	–	−0.604^**^ [−2.68]
profit _t−1_	0.852^***^ [3.66]	0.892^***^ [3.45]
expo_int _t−1_	0.006^***^ [2.61]	0.005^***^ [2.71]
fin_cons _t−1_	−0.078 [−1.46]	−0.095 [−1.55]
state_owner _t−1_	0.375 [0.92]	0.363 [1.09]
mana_eff _t−1_	0.774^***^ [6.14]	0.799^***^ [5.95]
gov_subs _t−1_	0.044^***^ [4.91]	0.053^***^ [5.04]
valuad_pro _t−1_	0.002 [1.17]	0.002 [1.11]
firm_age _t−1_	0.014 [1.27]	0.015 [1.38]
Constant term	Yes	Yes
Industry fixed-effects	Yes	Yes
Province fixed-effects	Yes	Yes
Observation	205,196	205,196
R-squared	0.181	0.179
F-statistics	624.5^***^	616.7^***^

*The t-statistics are in brackets*.

*** *p < 0.01 and*

** *p < 0.05*.

The left column considers the current pandemic discussion index in China (*china_pdi*_*t*_), and the right column uses the lagged pandemic discussion index in China (*china_pdi*_*t*−1_). Both measures of the pandemics-related uncertainty are negatively associated with corporate innovation. We observe that the current pandemic discussion index in China is statistically significant at the 1% level. In addition, the lagged pandemic discussion index in China is statistically significant at the 5% level.

We also use control variables in the fixed effects estimations. Similarly, we find that the operation profits (*profit*_*t*_), the total exports (*expo_int*_*t*_), the management efficiency (*mana_eff*_*t*_), and the government subsidies (*gov_subs*_*t*_) promote the level of corporate innovation. All of these indicators are found as statistically significant at the 1% level. The state ownership (*state_owner*_*t*_), the value-added productivity (*valuad_pro*_*t*_), and the firm age (*firm_age*_*t*_) are also positively related to corporate innovation; however, the related coefficients are statistically insignificant. Finally, we observe that financial constraints (*fin_cons*_*t*_) decrease the level of corporate innovation, but the related coefficients are also statistically insignificant.

### Findings of the Blundell–Bond Estimations: The Current Model

Table 5 reports the results of the system GMM estimations of Blundell and Bond (55) with the current controls. The dependent variable is the corporate innovation (*innov*_*t*_), and the lagged dependent variable is also included in the system GMM estimations. At this stage, the industry fixed-effects and the province fixed-effects are also included. In terms of diagnostics of the system GMM estimations, we find a significant first-order autocorrelation following the AR(1) test results. In addition, AR(2) test results indicate that there is no statistically significant second-order autocorrelation. Finally, the Sargan test results indicate no problem related to over-identifying restrictions of the system GMM estimations with the current model.

**Table 5:** Results of the Blundell–Bond estimations (current controls).

**Dependent variable: innov_**t**_**	**Full-sample**	
china_pdi _t_	−0.891^***^ [−6.69]	–
china_pdi _t−1_	–	−0.735^***^ [−3.81]
profit _t_	0.854^***^ [5.81]	0.882^***^ [5.48]
expo_int _t_	0.005^**^ [2.13]	0.005^**^ [2.35]
fin_cons _t_	−0.026 [−0.67]	−0.035 [−1.26]
state_owner _t_	0.398 [0.63]	0.369 [1.25]
mana_eff _t_	0.514^***^ [3.91]	0.532^***^ [4.38]
gov_subs _t_	0.071^***^ [4.62]	0.078^***^ [4.85]
valuad_pro _t_	0.003 [0.65]	0.003 [0.71]
firm_age _t_	0.013 [1.34]	0.014 [1.46]
Constant term	Yes	Yes
Lagged dependent variable	Yes	Yes
Industry fixed-effects	Yes	Yes
Province fixed-effects	Yes	Yes
AR(1)	(0.00)	(0.00)
AR(2)	(0.64)	(0.62)
Sargan test	(0.43)	(0.41)

*The t-statistics are in brackets and the probability values in parentheses*.

*** *p < 0.01 and*

** *p < 0.05*.

The left column considers the current pandemic discussion index in China (*china_pdi*_*t*_), and the right column focuses on the lagged pandemic discussion index in China (*china_pdi*_*t*−1_). Both measures of the pandemics-related uncertainty are negatively related to corporate innovation. Note that both the current and the lagged pandemic discussion index in China is statistically significant at the 1% level.

Similar to the fixed-effects estimations, we consider several control variables. We observe that the operation profits (*profit*_*t*_), the total exports (*expo_int*_*t*_), the management efficiency (*mana_eff*_*t*_), and the government subsidies (*gov_subs*_*t*_) promote the level of corporate innovation. At this point, the operation profits, the management efficiency, and the government subsidies are statistically significant at the 1% level. Note that the total exports are statistically significant at the 5% level.

In addition, the state ownership (*state_owner*_*t*_), the value-added productivity (*valuad_pro*_*t*_), and the firm age (*firm_age*_*t*_) are also positively related the corporate innovation; however, their coefficients are found as statistically insignificant. On the other hand, financial constraints (*fin_cons*_*t*_) decrease the level of corporate innovation. However, the related coefficients are also statistically insignificant.

### Findings of the Blundell–Bond Estimations: The Lagged Model

Table 6 provides the findings of the system GMM estimations of Blundell and Bond (55) with the lagged controls. The dependent variable is the corporate innovation (*innov*_*t*_), and the lagged dependent variable is also added in the system GMM estimations. In addition, the industry fixed-effects and the province fixed-effects are also included to the system GMM estimations. When we look at the diagnostics of the system GMM estimations, we observe a significant first-order autocorrelation, according to the AR(1) test results. Furthermore, AR(2) test findings show that there is no statistically significant second-order autocorrelation. Finally, according to the results of the Sargan test, there is no problem related to over-identifying restrictions of the system GMM estimations with the lagged model.

**Table 6:** Results of the Blundell–Bond estimations (lagged controls).

**Dependent variable: innov_**t**_**	**Full-sample**	
china_pdi _t_	−0.558^***^ [−6.97]	–
china_pdi _t−1_	–	−0.497^***^ [−3.49]
profit _t−1_	0.825^***^ [5.94]	0.855^***^ [5.22]
expo_int _t−1_	0.006^**^ [2.16]	0.006^**^ [2.40]
fin_cons _t−1_	−0.057 [−1.39]	−0.072 [−0.95]
state_owner _t−1_	0.357 [0.92]	0.345 [1.07]
mana_eff _t−1_	0.582^***^ [4.10]	0.633^***^ [4.29]
gov_subs _t−1_	0.054^***^ [3.95]	0.067^***^ [3.61]
valuad_pro _t−1_	0.002 [1.36]	0.002 [1.47]
firm_age _t−1_	0.011 [1.39]	0.011 [1.43]
Constant term	Yes	Yes
Lagged dependent variable	Yes	Yes
Industry fixed-effects	Yes	Yes
Province fixed-effects	Yes	Yes
AR(1)	(0.00)	(0.00)
AR(2)	(0.72)	(0.68)
Sargan test	(0.37)	(0.36)

*The t-statistics are in brackets and the probability values in parentheses*.

*** *p < 0.01 and*

** *p < 0.05*.

Like the previous estimations, the left column uses the current pandemic discussion index in China (*china_pdi*_*t*_), and the right column focuses on the lagged pandemic discussion index in China (*china_pdi*_*t*−1_). Both measures of the pandemics-related uncertainty are negatively associated with corporate innovation. Again, we observe that China's current and lagged pandemic discussion index is statistically significant at the 1% level.

Similar to the fixed-effects and the previous system GMM estimations, we use several control variables. We find that the operation profits (*profit*_*t*_), the total exports (*expo_int*_*t*_), the management efficiency (*mana_eff*_*t*_), and the government subsidies (*gov_subs*_*t*_) lead to a higher level of corporate innovation. At this stage, the operation profits, management efficiency, and government subsidies are statistically significant at the 1% level; however, the total exports are statistically significant at the 5% level.

Furthermore, the state ownership (*state_owner*_*t*_), the value-added productivity (*valuad_pro*_*t*_), and the firm age (*firm_age*_*t*_) increase the corporate innovation; however, their coefficients are statistically insignificant. On the other hand, the financial constraints (*fin_cons*_*t*_) reduce the Chinese corporate innovation level. Again the related coefficients are also statistically insignificant.

Overall, we find that the pandemics-related uncertainty harms corporate innovation. The firms' management efficiency, the government subsidies, the operation profits, and the total exports increase the corporate innovation of the Chinese firms.

## Concluding Remarks

The firms in developing countries need high value-added production, high-level technology, and higher productivity to sustain corporate innovation and thus economic performance. In this paper, we examine the effects of the pandemics-related uncertainty on corporate innovation in Chinese firms. We use the firm-level data in different industries and provinces in China. For this purpose, we consider the recent uncertainty measure of pandemics, so-called the PDI. The results from the fixed-effects estimations indicate the negative impact of the PDI on corporate innovation. We also observe that government subsidies, operation profits, and total exports positively affect corporate innovation. In addition, firms' management efficiency promotes corporate innovation. These results hold when we utilize the Blundell-Bond estimations to address potential endogeneity. Additional robustness analyses, such as considering the lagged PDI and the lagged controls, are also conducted. Consequently, the main results remain robust.

This paper provides novel and robust evidence on the negative impact of pandemics on China's corporate innovation behavior. These results provide several implications. These results show that government subsidies should be increased to promote corporate innovation, and increasing total exports is helpful to increase corporate innovation. Pandemics is negatively related to corporate innovation, but government support can decrease the operating costs, and this policy implication can increase corporate innovation. In developing economies, such as China, governments have more importance in supporting corporate innovation. It is also important to note that investments in human capital are an important channel to promote corporate innovation. Investments in human capital can increase managerial efficiency. Also, investments in human capital can increase professionalism in the workers.

Overall, firms can increase corporate innovation by technology, promoting production quality, increasing profits, and promoting competitiveness with exports. It is important to note that our findings are only limited to Chinese firms. At this stage, future papers can focus on other large developing economies, such as Brazil, India, and Russia, to analyse the determinants of corporate innovation. A special role can be given to pandemics-related indicators if the data will be available for the COVID-19 era.

## Data Availability Statement

Publicly available datasets were analyzed in this study. This data can be found here: Chinese Industrial Enterprise Database with the MOC List of Chinese firms.

## Author Contributions

CZ: writing the paper and estimations. YHu: writing the paper and methodology. LH: reviewing the paper and data collection. YHua: writing the paper and validation. All authors contributed to the article and approved the submitted version.

## Funding

The authors acknowledge the financial supports from the Philosophy & Social Science Fund of Tianjin City, China (project #: TJYJ20-012).

## Conflict of Interest

YH was employed by company Johnson & Johnson Medical (Shanghai) Ltd. The remaining author declares that the research was conducted in the absence of any commercial or financial relationships that could be construed as a potential conflict of interest.

## Publisher's Note

All claims expressed in this article are solely those of the authors and do not necessarily represent those of their affiliated organizations, or those of the publisher, the editors and the reviewers. Any product that may be evaluated in this article, or claim that may be made by its manufacturer, is not guaranteed or endorsed by the publisher.
